# Identification and Characterization of *als* Genes Involved in D-Allose Metabolism in Lineage II Strain of *Listeria monocytogenes*

**DOI:** 10.3389/fmicb.2018.00621

**Published:** 2018-04-04

**Authors:** Lu Zhang, Yan Wang, Dongxin Liu, Lijuan Luo, Yi Wang, Changyun Ye

**Affiliations:** State Key Laboratory of Infectious Disease Prevention and Control, National Institute for Communicable Disease Control and Prevention, Collaborative Innovation Center for Diagnosis and Treatment of Infectious Diseases, Chinese Center for Disease Control and Prevention, Beijing, China

**Keywords:** *Listeria monocytogenes*, lineage II, D-allose, metabolism, genes

## Abstract

*Listeria monocytogenes*, an important food-borne pathogen, causes listeriosis and is widely distributed in many different environments. In a previous study, we developed a novel enrichment broth containing D-allose that allows better isolation of *L. monocytogenes* from samples. However, the mechanism of D-allose utilization by *L. monocytogenes* remains unclear. In the present study, we determined the metabolism of D-allose in *L. monocytogenes* and found that lineage II strains of *L. monocytogenes* can utilize D-allose as the sole carbon source for growth, but lineage I and III strains cannot. Transcriptome analysis and sequence alignment identified six genes (*lmo0734* to *0739*) possibly related to D-allose metabolism that are only present in the genomes of lineage II strains. Recombinant strain ICDC-LM188 containing these genes showed utilization of D-allose by growth assays and Biolog phenotype microarrays. Moreover, *lmo0734* to *0736* were verified to be essential for D-allose metabolism, *lmo0737* and *0738* affected the growth rate of *L. monocytogenes* in D-allose medium, while *lmo0739* was dispensable in the metabolism of D-allose in *L. monocytogenes*. This is the first study to identify the genes related to D-allose metabolism in *L. monocytogenes*, and their distribution in lineage II strains. Our study preliminarily determined the effects of these genes on the growth of *L. monocytogenes*, which will benefit the isolation and epidemiological research of *L. monocytogenes*.

## Introduction

*Listeria monocytogenes* is a gram-positive foodborne pathogen of humans that causes listeriosis (Low and Donachie, 1997). Clinical symptoms include meningitis, septicemia, abortion, perinatal infections, and gastroenteritis. The elderly, newborns, pregnant women, and immunocompromised patients are more susceptible (Low and Donachie, 1997; Mead et al., 1999; Kathariou, 2002). Based on the serological reactions of somatic (O) and flagellar antigens (H), *L. monocytogenes* is divided into 13 serotypes, comprising 1/2a, 1/2b, 1/2c, 3a, 3b, 3c, 4a, 4ab, 4b, 4c, 4d, 4e, and 7. Previous study has been reported that a multiplex PCR assay has been developed to identify the serotype, which can separate four major serotype groups (Ward et al., 2008). Furthermore, 1/2b, 3b, 4b, 4d, 4e, and 7 belong to lineage I; serotypes 1/2a, 1/2c, 3a, and 3c belong to lineage II; while serotypes 4a and 4c belong to lineage III (Roberts et al., 2006). Some 4a, 4c, and atypical 4b strains were assigned to lineage IV based on sequence data analysis (Orsi et al., 2011). Lineage I strains are mostly isolated from human listeriosis cases, lineage II mostly exist in food and the environment, and lineage III and IV are rare and mainly found in animal hosts (Liu, 2008). Nevertheless, Zilelidou et al. (2015) have reported that multiply *L. monocytogenes* strains could isolate from a single food sample, suggesting that highly invasive *L. monocytogenes* strains might show stronger growth than low or modestly invasive strains. However, studies did not suggest a link between the competitive advantage and strain origin, serotype, or sequence type (Zilelidou et al., 2015, 2016).

*L. monocytogenes* is ubiquitous in the environment and is often isolated from contaminated environments and foods. Notably, *L. monocytogenes* has the ability to grow at refrigeration temperatures and survive at high salt concentrations, making it difficult to control (Zhang, 2007). *L. monocytogenes* also can adhere to the surfaces of food processing equipment and resist adverse conditions. It has been reported that *L. monocytogenes* was persistently isolated from raw pork in open markets in China (Luo et al., 2017). *L. monocytogenes* is aerobic, or facultatively anaerobic under some conditions. *L. monocytogenes* can metabolize different carbon sources, including D-glucose. Strains can utilize D-glucose to form lactate, acetate, and acetoin when they are grown aerobically, whereas, acetoin is not formed during anaerobic growth (Pine et al., 1989). In addition, cellobiose, fructose, mannose, galactose, lactose, salicin, maltose, dextrin, and glycerol can also be utilized to produce acid. The growth rate of *L. monocytogenes* increases if fermentable sugars are present (Siddiqi and Khan, 1982).

D-Allose, an aldohexose, is a rare monosaccharide in nature and its physiological functions are varied. D-allose inhibits tumor cell multiplication and active oxygen production (Murata et al., 2003). D-allose is used in a novel enrichment broth to improve the isolation of *L. monocytogenes* and reduce the growth of non-target organisms (Liu et al., 2017). However, the details of the utilization of D-allose in *L. monocytogenes* are unknown. D-allose has been reported to be a carbon source for *Escherichia coli* (Gibbins and Simpson, 1964). It could be converted to fructose-6-phosphate via D-allose-6-phosphate and D-allulose-6-phosphate in *Aerobacter aerogenes* and *E. coli* K12 (Gibbins and Simpson, 1964; Kim et al., 1997). Two operons are involved in D-allose metabolism in *E. coli*, one contains the *alsI* gene, which encodes allose 6-phosphate isomerase, and the other consists of six contiguous genes, *alsR, B, A, C, E*, and *K*. *alsR* is a negative regulator for the operon. *alsB, A*, and *C* comprise the transport system of D-allose, *alsB* encodes a D-allose-binding protein, *alsA* encodes an ATP-binding component, and *alsC* encodes a transmembrane protein. The allulose 6-phosphate epimerase is encoded by *alsE* and *alsK* is thought to encode allokinase. Furthermore, the two operons are adjacent in the genome of *E. coli* K12 (Poulsen et al., 1999). In *L. monocytogenes*, operons comprising the genes involved in D-allose are unknown. In this study, we investigated the utilization of D-allose in *L. monocytogenes* and analyzed the associated genes for D-allose metabolism.

## Methods and materials

### Strains, plasmids, media, and culture conditions

*L. monocytogenes* strains EGD-e and ICDC-LM188 were used as D-allose utilizing and D-allose non-utilizing control strains, respectively. A total of 278 *L. monocytogenes* strains, isolated from different areas and sample sources in China, were used for the D-allose utilization assay in this study (Supplementary Table 1). Lineage I and III strains (Table 1) were used for gene function analysis. These strains originated from American Type Culture Collection (ATCC, USA) or were isolated in China and stored in State Key Laboratory of Infectious Disease Prevention and Control, China CDC at −80°C (Table 1). Plasmid PIMK2 was used to construct the gene expression vectors (Table 1; Lauer et al., 2002). Bacterial cells were cultured in Brain Heart Infusion (BHI) Broth (BD, USA) with shaking at 220 rpm. Modified Welshimer's broth (MWB) medium was used in the growth assay (Premaratne et al., 1991), and Luria-Bertani (LB) medium (Oxoid, UK) was used in the transcriptome analysis.

**Table 1:** Plasmids and strains used in this study.

**Strains or plasmids**	**Genotypes**
**PLASMIDS**	
pIMK2	pIMK rrnB T1 terminators Phelp
pAL1	pIMK2 *lmo0734 0735 0736 0737 0738 0739*
pAL2	pIMK2 *lmo0734 0735 0736 0737 0738*
pAL3	pIMK2 *lmo0734 0735 0736 0737 0739*
pAL4	pIMK2 *lmo0734 0735 0736 0738 0739*
pAL5	pIMK2 *lmo0734 0735 0737 0738 0739*
pAL6	pIMK2 *lmo0734 0736 0737 0738 0739*
pAL7	pIMK2 *lmo0735 0736 0737 0738 0739*
**STRAINS (SEROTYPE)**	
EGD-e (1/2a)	*lmo0734, 0735, 0736, 0737, 0738, 0739*
ICDC-LM188 (1/2b)	None
RS801 (1/2b)	ICDC-LM188::pAL1
RS802 (1/2b)	ICDC-LM188::pAL2
RS803 (1/2b)	ICDC-LM188::pAL3
RS804 (1/2b)	ICDC-LM188::pAL4
RS805 (1/2b)	ICDC-LM188::pAL5
RS806 (1/2b)	ICDC-LM188::pAL6
RS807 (1/2b)	ICDC-LM188::pAL7
ATCC 19114 (4a)	None
RS19114 (4a)	ATCC19114::pAL1
ATCC 19115 (4b)	None
RS19115 (4b)	ATCC19115::pAL1
ACTC 19116 (4c)	None
RS19116 (4c)	TCC19116::pAL1
ATCC 19117 (4d)	None
RS19117 (4d)	ATCC19117::pAL1
ATCC 19118 (4e)	None
RS19118 (4e)	ATCC19118::pAL1

### D-allose utilization and growth curve analysis

Single colonies of *L. monocytogenes* strains EGD-e and ICDC-LM188 were incubated in BHI medium with constant shaking at 220 rpm overnight at 37°C, and then a 1% volume of the BHI cultures were transferred separately into BHI medium, and shaken at 220 rpm at 37°C until the OD_600_ values reached 0.6. The strains were then diluted 1:100 into 0.2% D-allose or D-glucose MWB medium (MWB medium containing 2 g/L D-allose or D-glucose as the sole carbohydrate). The strains were grown in a Bioscreen C microbiology reader (Growth Curves Ltd, Helsinki, Finland) at 37°C with shaking. The OD_600_ was monitored at 30 min intervals for 24 h. To obtain the maximum growth rate for each strain (Pontinen et al., 2017), the OD_600_ data were fitted to growth curves using GraphPad Prism software (GraphPad Software Inc., San Diego, CA, USA).

To test the universality of D-allose utilization in *L. monocytogenes*, a single colony of each of 278 *L. monocytogenes* strains was cultured in BHI medium at 37°C with shaking at 220 rpm overnight, and 1% volumes of the cultures were transferred into BHI with shaking 220 rpm at 37°C until the OD_600_ value reached 0.6. Subsequently, the strains were diluted 1:100 into 0.2% D-Allose MWB medium, and the cultures were shaken at 220 rpm for 24 h at 37°C. Finally, the growth status of the bacterial cells were detected.

### Transcriptome analysis and D-allose metabolism related gene detection

*L. monocytogenes* EGD-e was selected as the reference for the transcriptome analysis. One colony of EGD-e was incubated in BHI medium at 37°C with shaking at 220 rpm overnight. Then, 1% volumes of the culture were respectively transferred into 0.2% D-allose and D-glucose LB medium at 37°C with shaking at 220 rpm until the OD_600_ value reached 0.6. The bacteria were then harvested by centrifugation and total RNA was extracted using the Trizol method (Rio et al., 2010). The extracted RNA was used for RNA sequencing with BGISEQ-500, according to the sequencing procedure used by the Beijing Genomics Institute (BGI, China). After filtering out low quantity sequences, clean reads were generated and mapped to the reference genome using HISAT and Bowtie2 tools (Langmead et al., 2009; Kim et al., 2015). The Poisson distribution method was used to analyze differentially expressed genes (DEGs). Clustering analysis of DEGs was performed with cluster and java Treeview software (Michael Eisen, Stanford University, Stanford, CA). Gene Ontology (GO) (http://www.geneontology.org/) annotation was performed for the screened DEGs. Six pairs of specific primers were designed to verify DEGs based on the results of the transcriptome analysis (Supplementary Table 2). DNAs of above 278 *L. monocytogenes* strains were isolated by boiling, detected by PCR using the specific primers, and confirmed by agarose gel electrophoresis.

### Plasmids construction and electro-transformation

**Figure 1:**
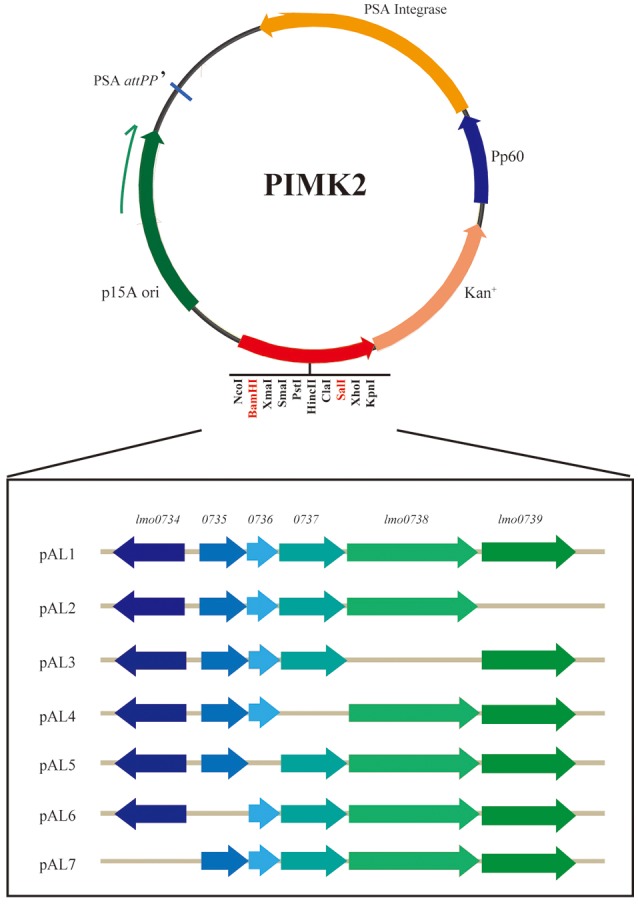
Schematic diagram of the expression vector construction. The gene sequences were cloned into restriction endonuclease recognition sites *Bam*HI and *Sal*I. Each recombinant plasmid lacked one gene in the cluster. The direction of transcription of each is marked by an angled arrow.

To construct the D-allose related gene expression vectors, the DNA fragments, being obtained by PCR using the specific primers (Table 2), were purified from agarose gel electrophoresis using a DNA Gel Extraction kit (Takara, Shiga, Japan). The purified products were cloned into the *Sal*I and *Bam*HI sites of vector pIMK2 to form plasmids pAL1 to pAL7 (Figure 1) via In-Fusion or T4 ligase Cloning. Different lineage strains (Table 1) were used to prepare competent cells (Camilli et al., 1990). Plasmid pAL1 was electrotransformed into competent cells of the strains of all lineages except lineage II, and plasmids pAL2, pAL3, pAL4, pAL5, pAL6, and pAL7 were respectively introduced into competent cells of *L. monocytogenes* strain ICDC-LM188 (Table 1). The electroporation conditions were 2.5 KV, 400 Ω, 25 μF. Afterwards, the strains were grown on BHI plates containing 50 μg/ml kanamycin in a 37°C incubator for 24 h. The clones were detected by PCR using the specific primers (Supplementary Table 2).

**Table 2:** Primers used to construct the expression vectors.

**Plasmids**	**Primers (5′-3′)**
pAL1	AL1 P1 F
	CGCGGATCCTTAAATTGAGTCTTTTAAAGATAATTCAACGGGAACAATCTCTG
	AL1 P1 R
	CGCGTCGACTCAGTAGTTCAAATCATTTCCATTAG
pAL2	AL2 P1 F
	CCATGGAAAAGGATCCTTAAATTGAGTCTTTTAAAGATAATTCAACGGGAACAATCTCTG
	AL2 P1 R
	AGTTGCTAGGTAACGATGTCTTAATTTTCGC
	AL2 P2 F
	GCGAAAATTAAGACATCGTTACCTAGCAACT
	AL2 P2 R
	ACCCCCTCGAGTCGACTTAAAGTACTGGTGTTAAAAGCGTAT
pAL3	AL3 P1 F
	CCATGGAAAAGGATCCTTAAATTGAGTCTTTTAAAGATAATTCAACGGGAACAATCTCTG
	AL3 P1 R
	TTGCAAATTAAACATTTACGATTTCTGCTTGCCATTCTAAAGTACGTATAAGGT
	AL3 P2 F
	AAGCAGAAATCGTAAATGTTTAATTTGCAAAAAGGTTTTCCAG
	AL3 P2 R
	ACCCCCTCGAGTCGACTCAGTAGTTCAAATCATTTCCATTAG
pAL4	AL4 P1 F
	CCATGGAAAAGGATCCTTAAATTGAGTCTTTTAAAGATAATTCAACGGGAACAATCTCTG
	AL4 P1 R
	TTTTTGATAATCCATTTAATCATTTTCATCTTCAATTCTAGCAATCATTTCGACGCG
	AL4 P2 F
	GATGAAAATGATTAAATGGATTATCAAAAACTAGCTAAAGAG
	AL4 P2 R
	ACCCCCTCGAGTCGACTCAGTAGTTCAAATCATTTCCATTAG
pAL5	AL5 P1 F
	CCATGGAAAAGGATCCTTAAATTGAGTCTTTTAAAGATAATTCAACGGGAACAATCTCTG
	AL5 P1 R
	TACTTCTTGCTCCATTCATCGTAATTCCTCCATCTTTTCTTG
	AL5 P2 F
	GAGGAATTACGATGAATGGAGGCAAGAAGTAACACGACTAAT
	AL5 P2 R
	ACCCCCTCGAGTCGACTCAGTAGTTCAAATCATTTCCATTAG
pAL6	AL6 P1 F
	CCATGGAAAAGGATCCTTAAATTGAGTCTTTTAAAGATAATTCAACGGGAACAATCTCTG
	AL6 P1 R
	AATAGCAATTTTCATATGAAAAAAACATCCATAAAAGATATCGC
	AL6 P2 F
	GGATGTTTTTTTCATATGAAAATTGCTATTGGAAATGATCATGTTGG
	AL6 P2 R
	ACCCCCTCGAGTCGACTCAGTAGTTCAAATCATTTCCATTAG
pAL7	AL7 F
	CGCGGATCCATGAGCAAAAAACTGATTTGTCCTTCTAT
	AL7R
	CGCGTCGACTCAGTAGTTCAAATCATTTCCATTAG

### Growth phenotype and curve analysis

The positive clones were incubated in BHI medium at 37°C with shaking at 220 rpm overnight, 1% volumes of cultures were transferred into BHI medium with shaking at 37°C until the OD_600_ value reached 0.6. Afterwards, 1% volumes of inoculum were cultured in 0.2% D-allose MWB medium with shaking 220 rpm at 37°C for 24 h. The cells growth status was monitored (Zwietering et al., 1990; Markkula et al., 2012). Biolog phenotype microarrays (Biolog, USA) were used to analyze the utilization of D-allose by *L. monocytogenes* strain ICDC-LM188 and its recombinant strains (Miller and Rhoden, 1991). Based on the manufacturer's protocol for *Listeria*, the Biolog 96-well micro-plates PM1 and PM2a were used to test carbohydrate metabolism; plate PM2a contains D-allose. Clones were inoculated into 20 ml 1 × Buffer IF-0a, and the turbidity of the suspension was checked until 81% light transmittance was achieved. Then, 1.76 ml of the cell suspension was transferred into 22.24 ml PM1 and PM2a inoculating fluid, and 100 μl/well of the cell suspension was inoculated in 96-well micro-plates, which were incubated in an OmniLog instrument at 37°C for 24 h. Growth curve analysis of the recombinant strains was carried out according to a previous description (Pontinen et al., 2017) using the Bioscreen C microbiology reader (Growth Curves Ltd., Helsinki, Finland.) at 37°C, 0.2% D-allose medium was used as the broth.

## Results

### D-allose utilization and growth assay

**Figure 2:**
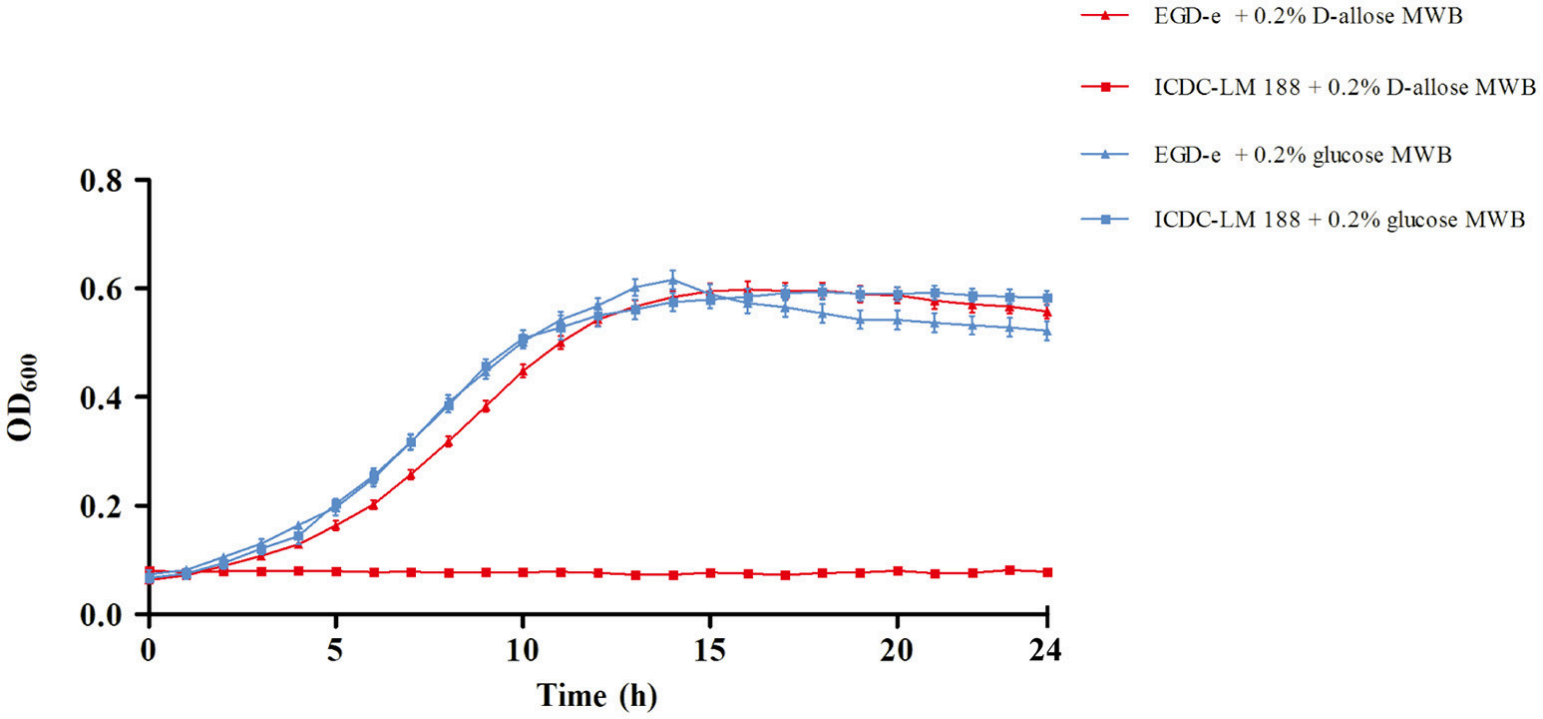
Growth curve of *L. monocytogenes* EGD-e and ICDC-LM188. The red line shows growth in 0.2% D-allose modified Welshimer's broth (MWB) medium, the blue line shows growth in 0.2% D-glucose MWB medium. Triangles represent EGD-e and squares represent ICDC-LM188.

In our previous study, D-allose was discovered to be a carbon source for *L. monocytogenes* growth, and *L. monocytogenes* could grow in the novel *Listeria* Allose Enrichment Broth (LAEB). In addition to D-allose, tryptone, peptone, and Lab-lemco powder could contributed a small amount of carbon source and energy in the LAEB medium (Liu et al., 2017). In this study, to identify D-allose utilization in the growth of *L. monocytogenes*, MWB medium was used (Premaratne et al., 1991). D-glucose was replaced by D-allose in MWB medium as the sole carbon and energy source for growth. *L. monocytogenes* strains EGD-e and ICDC-LM188 were used as experimental references, because EGD-e could grow well in LAEB medium, while ICDC-LM188 could not (Liu et al., 2017). Growth rates were calculated (Zwietering et al., 1990; Markkula et al., 2012) and were similar between EGD-e and ICDC-LM 188 in 0.2% D-glucose MWB medium. Moreover, there was no obvious difference in the growth of EGD-e between D-glucose and D-allose medium (Figure 2). By contrast, no growth of ICDC-LM 188 was observed in the 0.2% D-allose MWB medium (Figure 2). Thus, ICDC-LM188 could not utilize D-allose as carbon and energy source.

In addition, 278 experimental strains were cultured as above to verify their D-allose utilization. The result showed that 171 strains belonging to lineage II could grow in D-allose MWB medium; however, the other 107 strains, including 91 strains of lineage I and 16 strains of lineages III and IV, could not (Table 3). The result was analyzed using Fisher's test, which revealed that there was a correlation (Pearson *r* = 0.7071, *P* < 0.001) between the lineages and D-allose utilization.

**Table 3:** D-allose utilization in *L. monocytogenes* strains.

**Growth in D-allose medium**				
		**+**	**−**	**Total**
	I	0	91	91
Lineage	II	171	0	171
	III	0	16	16
Total		171	107	278

### Transcriptome analysis and D-allose metabolism related gene detection

Using RNA-Seq technology, more than 23 million clean reads were acquired after filtering out low quality reads. The transcriptomes were successfully sequenced and deposited in GenBank (Accession No. SRR6281666 and SRR6281667). According to the quality control and expression level calculated using the RSEM algorithm and the fragments per kilobase of transcript per million mapped reads (FPKM) method (Li and Dewey, 2011), the differential expression of 15 genes were upregulated and 13 genes were downregulated in D-allose utilizing group compared with the D-glucose utilizing group. Moreover, pathway enrichment analysis of the DEGs was performed based on the Kyoto Encyclopedia of Genes and Genomes (KEGG) database, and the functions of the DEGs were classified as nucleotide metabolism and carbohydrate metabolism in the secondary pathway of KEGG (Supplementary Table 3).

**Figure 3:**
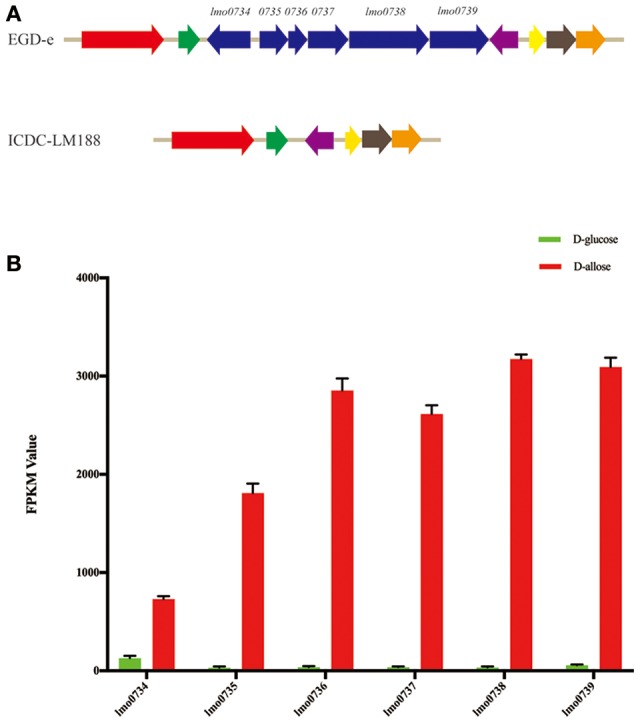
Genome analysis and gene expression levels. **(A)** The cluster of whole-genome alignment between *L. monocytogenes* EGD-e and ICDC-LM188. The same color angled arrows represent the same genes. Six genes of EGD-e were found to be absent in ICDC-LM188 (between the green and purple angled arrows). **(B)** Comparison of the expression level comparisons of five different genes between D-glucose and D-allose (*P* < 0.001). FPKM, fragments per kilobase of transcript per million mapped reads.

Subsequently, whole-genome alignment of different lineage strains was conducted and a gene cassette (*lmo0734* to *0739*) of strain EGD-e was observed to be absent in *L. monocytogenes* lineage I, III, and IV strains (Figure 3A). This specific gene cassette was highly expressed under D-allose utilization conditions (Figure 3B). DNA was extracted from 278 experimental strains, including different lineages, to verify the presence of the genes in the different lineages. All strains of lineage II contained the gene cassette; however, it was not present in any of the other lineages. Notably, only the strains of lineage II could utilize D-allose as a carbon source for growth. These results were analyzed using Fisher's test, which showed that there was a correlation between the six genes and D-allose utilization in *L. monocytogenes* (Pearson *r* = 0.7071, *P* < 0.001).

### Growth and biolog characterization of recombinant strains

D-allose utilization of the recombinant strains was verified using a growth assay (Table 1). D-allose supported the growth in different lineage strains containing pAL1 (containing all six genes; Supplementary Figure 1A). This revealed that the specific gene cassette was involved in D-allose metabolism in different lineage strains of *L. monocytogenes*. To further identify the key genes in the D-allose metabolism of *L. monocytogenes*, plasmids pAL2, pAL3, pAL4, pAL5, pAL6, and pAL7 were constructed according to the schematics shown in Figure 1, and were transferred separately into strain ICDC-LM188. Strains RS802, RS803, and RS804 grew on D-allose medium; however, strains RS805, RS806, and RS807 did not (Supplementary Figure 1B). This revealed that genes *lmo0734, lmo0735*, and *lmo0736* were essential for D-allose metabolism of *L. monocytogenes*.

**Figure 4:**
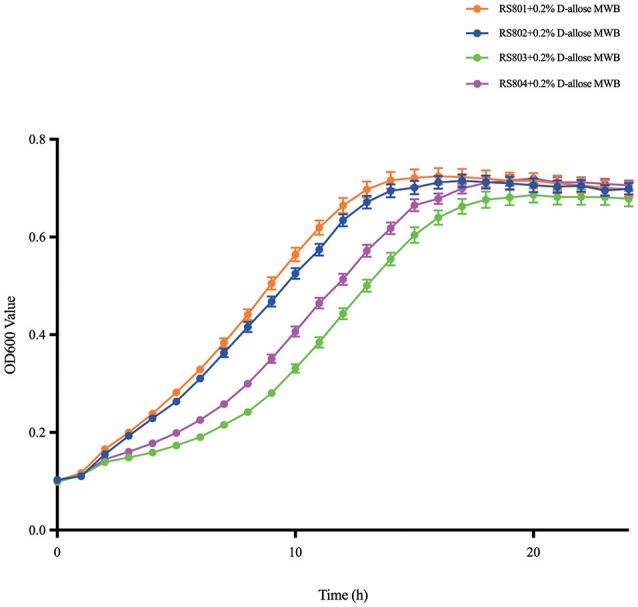
Growth curve of RS801, RS802, RS803, and RS804. Graph showing growth in 0.2% D-allose modified Welshimer's broth (MWB). Orange line: RS801; blue line: RS802; green line: RS803; purple line: RS804.

The recombinant strains RS801, RS802, RS803, and RS804 showed different growth rates in MWB medium with 0.2% D-allose (Figure 4). The growth curves were analyzed in Graphpad Prism, the specific growth rates were calculated (Table 4). According to *t*-test analysis (Zwietering et al., 1990; Pontinen et al., 2017), the growth rates of RS801 and RS802 were similar, while RS803 and RS804 were similar. Moreover, the growth rates of RS801 and RS802 were higher than those of RS803 and RS804 (*P* < 0.01).

**Table 4:** Growth rates of recombinant strains in D-allose.

**Strains**	**Growth rate ± *SD* (OD600 units/h)**
RS801	0.1504 ± 0.006
RS802	0.1486 ± 0.005
RS803	0.1055 ± 0.005
RS804	0.1082 ± 0.004

**Figure 5:**
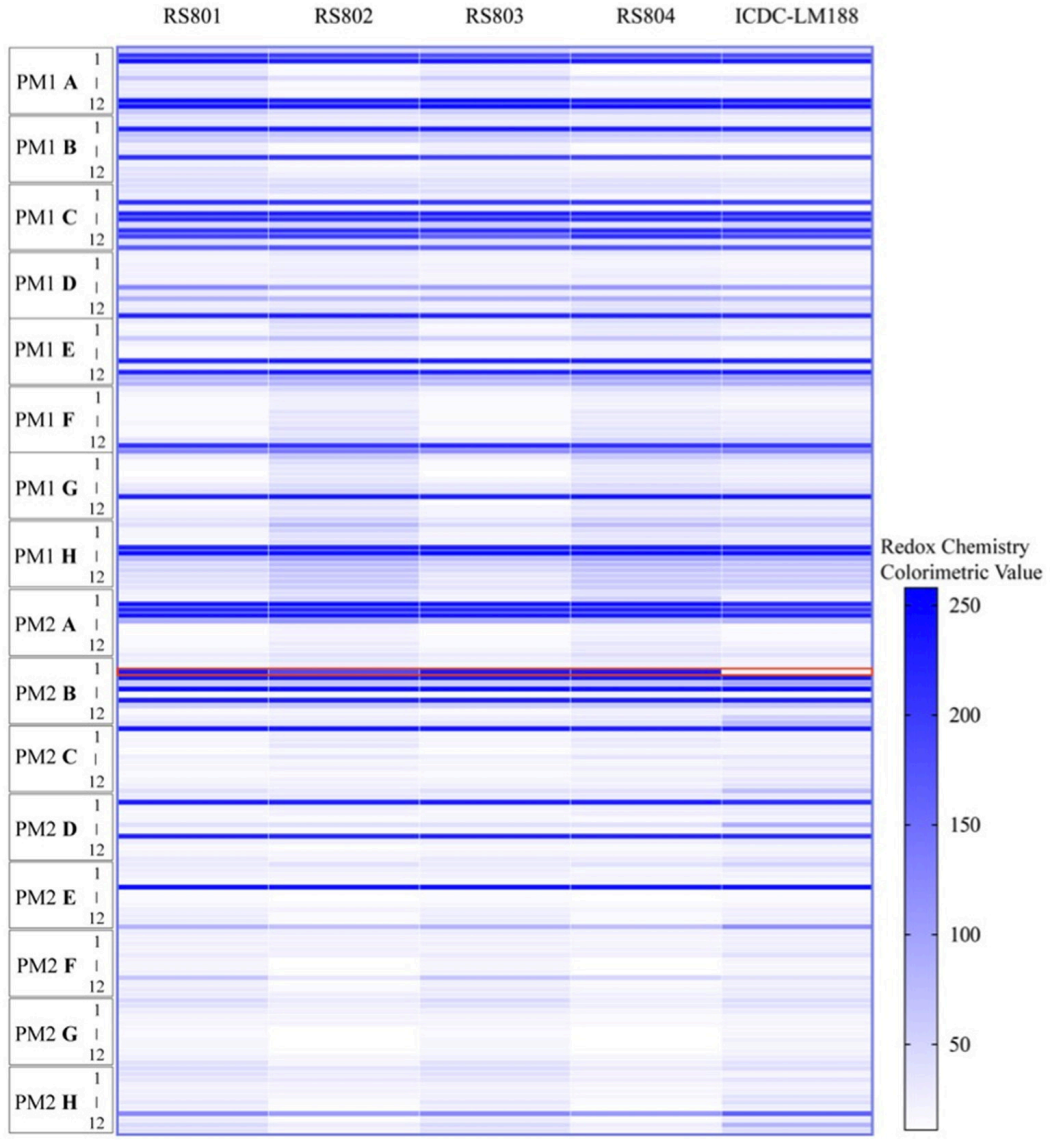
Phenotype microarray analysis of the utilization of carbohydrate by ICDC-LM188, RS801, RS802, RS803, and RS804. All plates were incubated at 37°C for 24 h. PM represent a PM plate, A–H and 1–12, respectively, represent the column and row of the PM plate. Each horizontal line in the heat map shows a type of carbohydrate metabolism. The red border marking the line represents the utilization of D-allose.

Biolog phenotype microarrays showed that recombinant strains RS801, RS802, RS803, and RS804 could grow on D-allose basal broth while ICDC-LM188 could not (Figure 5). In addition to metabolism of D-allose, the recombinant strains and ICDC-LM188 showed similar utilization of other carbohydrates. This revealed that the six genes (*lmo0734-0739*) represented a specific cassette involved in the carbohydrate metabolism of D-allose.

## Discussion

*L. monocytogenes* has been described as a resistant and ubiquitous bacterium that can survive in harsh conditions. Under nutrient-limited conditions, some subgroup lineage II strains can grow faster than some subgroups of lineage I strains (Tang et al., 2015), and most strains of lineage II were reported to be isolated more frequently from food samples than from patients (Chenal-Francisque et al., 2011; Montero et al., 2015). Some lineage II strains have been reported to form biofilms to adapt to harsh environments (Wong, 1998; Møretrø and Langsrud, 2004). In the previous study of LAEB, we found that some *L. monocytogenes* strains could utilize D-allose for growth (Liu et al., 2017). In the present study, we further confirmed that only strains of lineage II could utilize D-allose as a carbon source, and the other lineages could not, including 91 lineage I strains and 17 lineage III strains. This suggested the presence of special carbohydrate metabolisms in different lineage strains, which might play important roles in the growth and isolation of *L. monocytogenes* in different environments. The hypothesis needs to be studied further in the future.

In our study, a specific gene cassette associated with D-allose was present in lineage II strains, which allowed them to utilize D-allose. The cassette has been reported to be unique to lineage II and atypical 4b, but is absent in other lineages (Doumith et al., 2004; Milillo et al., 2009; Lee et al., 2012). In particular, *lmo0737* is regarded as a specific gene to identify lineage II (Ward et al., 2008). Additionally, the present of the specific gene cassette has been reported to have no impact on invasion and growth in nutrient-deprived conditions in knockout mutants of EGD-e (Milillo et al., 2009). Nonetheless, in this study, we verified that the gene cassette presents in lineage II strains and is related to D-allose metabolism.

Gene *lmo0734* is defined as a Lac I regulator in genome of EGD-e, and it is a member of the D-allose metabolism operon of *L. monocytogenes*. Transcriptional fusions in the *als* operon were Lac^+^ in the original background in D-allose metabolism of *E. coli* K12 (Kim et al., 1997). Genes *lmo0735* and *lmo0736* are regarded to have *alsE* and *alsI* functions. The three genes have been reported to be essential in D-allose metabolism of *L. monocytogenes*. In previous studies, *alsE* was required in D-allose metabolism of *E. coli* K12 and *A. aerogenes*, while *alsI* proved to be essential in *A. aerogenes* and dispensable in *E. coli K12* (Gibbins and Simpson, 1964; Kim et al., 1997; Poulsen et al., 1999). Gene *lmo0737* was annotated as a hypothetical protein belonging to a member of TIM phosphate binding superfamily. Phosphorylase kinase is one of the TIM family (Nagano et al., 1999). D-allose is phosphorylated to D-allulose by the product of *alsK*, a phosphorylase kinase. Gene *lmo0737* might be the equivalent to *alsK*, being responsible for phosphorylation for D-allose. Gene *lmo0738* is annotated as encoding a PTS beta-glucoside transporter subunit IIABC in EGD-e, which has the same function as the products of *alsA, B*, and *C*, namely transferring D-allose across the membrane. Genes *lmo0737* and *lmo0738* have been reported to be dispensable in D-allose metabolism in other bacteria. Nonetheless, absence of *lmo0737* and *lmo0738* decreased the growth rate in D-allose MWB medium. The requirement for *lmo0734* to *lmo0736* and the dispensability of *lmo0738* to *lmo0739* suggested that *lmo0734* to *lmo0736* are irreplaceable, while other genes might compensate for the activities of *lmo0737* and *lmo0738*. Interestingly, ribose has been reported as an analog of D-allose, and D-allose can be metabolized via some proteins in the D-ribose metabolic pathway (Kim et al., 1997). Gene *lmo0739* encodes 6-phospho-beta-glucosidase in *L. monocytogenes*. Currently, there are no studies to indicate 6-phospho-beta-glucosidase plays a role in D-allose metabolism. In addition, *lmo0739* is absent in genome of *Listeria ivanovii* PAM55, which can utilize D-allose (Liu et al., 2017).

In the transcriptome analysis, there were other DEGs in addition to genes *lmo0734* to *lmo0739*. Among the downregulated genes, genes *lmo0096* and *lmo0097* mainly participate in mannose metabolism. D-allose metabolism is a part of mannose metabolism (KEGG Pathway map00051). D-allose metabolism might have an affect on mannose metabolism. Genes *lmo2761* to *lmo2765* have functions in D-cellobiose metabolism, which is associated with D-glucose (KEGG Pathway map00050), which is isomer of D-allose. However, a clear relationship between D-allose and D-cellobiose metabolism has not been demonstrated. As for the correlation between these genes and D-allose metabolism, it need to be studied in future.

## Conclusion

Our previous study demonstrated that the utilization of D-allose in a novel enrichment broth could increase the isolation rate of *L. monocytogenes*. The present study firstly proposes D-allose metabolism in *L. monocytogenes* and identified the key genes involved in this metabolism. We determined the distribution of these genes in different lineage strains. We provide preliminarily evidence for the utilization of D-allose in the novel enrichment broth for *L. monocytogenes* lineage II strains. Our results will serve as a reference for the optimization of novel broth (LAEB) to increase the isolation of other lineages strains in the future and for further research into the carbohydrate metabolism of *L. monocytogenes*.

## Author contributions

LZ: Designed the project, analyzed data, and wrote manuscript; DL, LL, and YaW: Analyzed data; YiW and CY: Carried out the experiments.

### Conflict of interest statement

The authors declare that the research was conducted in the absence of any commercial or financial relationships that could be construed as a potential conflict of interest.
