# Supplementary material for: Identification and Characterization of als Genes Involved in D-Allose Metabolism in Lineage II Strain of Listeria monocytogenes
Source: Front Microbiol. 2018 Apr 4;9:621. doi: 10.3389/fmicb.2018.00621 (PMC5893763; doi:10.3389/fmicb.2018.00621)
Supplement: Supplementary file 1 [file Table1.DOCX]

**Supplementary Table 1.** **Information of 278 experimental strains**

| Information | Classification | Total |
| --- | --- | --- |
| Areas | Anhui(17), Beijing(92), Chongqing(2), Fujian(10), Guangdong(4), Henan(8), Hubei(9), Jiangsu(5), Jilin(1), Shandong(1), Shanghai(33), Shanxi(3), Sichuan(75), Yunnan(1), Zhejiang(17) | 15 |
| Years | 2000(4), 2001(12), 2002(9), 2003(6), 2004(17), 2005(14), 2006(5), 2007(20), 2008(17), 2009(11), 2010(14), 2011(7), 2012(3), 2014(58), 2015(78), 2016(1), unclear (2) | 17 |
| Sample sources | Aquatic products(19), Bean products(1), Beef(23), Beef booth(1), Chicken(30), Dissection booth(1), Duck(1), Environments(30), Frog-eel-loach booth(2), Frozen chicken(3), Frozen meet booth(2), Frozen pork(1), Fruit(1), Haggis restaurant(4), Ice cream(4), Intestinal content(1), Meat grinding shop(4), Mutton(3), Mutton shop(1), Patients(9), Pork(80), Pork booth(6), Rabbit leg(1), Ready-to-eat food(19), Ready-to-eat shop(11), Slaughterhouse(1), Vegetable(3), unclear (16) | 28 |
| Sequence Type (ST) | 1(10), 2(6), 3(3), 5(4), 6(2), 7(8), 8(17), 9(18), 11(4), 12(3), 14(4), 19(2), 34(1), 35(3), 59(4), 83(1), 87(44), 91(2), 101(6), 120(4), 121(23), 122(8), 123(4), 124(1), 145(3), 155(18), 177(1), 196(3), 199(4), 288(1), 295(3), 297(2), 299(10), 300(1), 301(1), 302(2), 303(1), 304(2), 305(1), 306(1), 307(7), 309(1), 310(1), 311(2), 312(1), 330(1), 356(1), 372(1), 378(1), 381(1), 412(1), 429(3), 451(1), 504(1), 515(1), 588(2), 705(1), 1001(1), 1002(4), 1003(6) | 60 |
| Clonal Complex (CC) | CC1(14), CC2(9), CC3(3), CC5(4), CC6(3), CC7(14), CC8(23), CC9 (39), CC11(4), CC14(6), CC19(3), CC59(4), CC87(46), CC101(6), CC121(23), CC124(1), CC131(11), CC155(25), CC177(1), CC193(3), CC199(4), CC288(5), CC412(1), CC451(1), CC475(1), CC218(2), ST300(1), ST301(1), ST307(7), ST429(3), ST1002(4), ST1003(6) | 32 |
