# Supplementary material for: Identification and Characterization of als Genes Involved in D-Allose Metabolism in Lineage II Strain of Listeria monocytogenes
Source: Front Microbiol. 2018 Apr 4;9:621. doi: 10.3389/fmicb.2018.00621 (PMC5893763; doi:10.3389/fmicb.2018.00621)
Supplement: Supplementary file 2 [file Table2.DOCX]

**Supplementary Table 2.** **Primers to verify differentially expressed genes**

| Gene | Primer (5’-3’) | Length |
| --- | --- | --- |
| *lmo0734* | F: CTGAAGACGATTGCCTGCT  R: TGGTGGATGGGTTGATTG | 300 bp |
| *lmo0735* | F: CAAAGCTGGAAGCGATGTTTTTCAT  R: TAATGCGGATGTTCCTAGCACAAAC | 523 bp |
| *lmo0736* | F: ATGAAAATTGCTATTGGAAATGATC  R: ATTTTCATCTTCAATTCTAGCAATC | 441 bp |
| *lmo0737* | F: GGCTTCAAGGACTTACCC  R: ATCGCCCAACACTTTCTC | 320 bp |
| *lmo0738* | F: CTCGGACATAGCAAAAGGGAC  R: GGCAAATCATCGGCGTTA | 494 bp |
| *lmo0739* | F: AAACGGCACTATCGAAACAGCTTT  R: AATAAAGTTTTGCTCGTTGAAAGG | 863 bp |

The annealing temperature of all primers was 54 ℃.
