# Supplementary material for: Identification and Characterization of als Genes Involved in D-Allose Metabolism in Lineage II Strain of Listeria monocytogenes
Source: Front Microbiol. 2018 Apr 4;9:621. doi: 10.3389/fmicb.2018.00621 (PMC5893763; doi:10.3389/fmicb.2018.00621)
Supplement: Supplementary file 3 [file Table3.DOCX]

**Supplementary Table 3. The FPKM of differentially expressed genes between D-glucose and D-allose**

| Gene ID | Gene  Length | | Average FPKM (Glucose) | Average FPKM (Allose) | log2 Ratio (Allose/Glucose) | Up/Down Regulation (Allose/Glucose) | Secondary pathway in KEGG |
| --- | --- | --- | --- | --- | --- | --- | --- |
| lmo0096 | | 966 | 2094.17 | 742.16 | -1.441913554 | Down | carbohydrate metabolism |
| lmo0097 | | 807 | 1905.41 | 611.165 | -1.540842101 | Down | carbohydrate metabolism |
| lmo0098 | | 912 | 3213.05 | 1057.995 | -1.523904874 | Down | carbohydrate metabolism |
| lmo1831 | | 630 | 630.065 | 124.075 | -2.352621963 | Down | nucleotide metabolism |
| lmo1832 | | 702 | 652.125 | 144.74 | -2.159676201 | Down | nucleotide metabolism |
| lmo1833 | | 915 | 685.055 | 153.055 | -2.16245193 | Down | nucleotide metabolism |
| lmo1834 | | 765 | 624.035 | 139.845 | -2.156130076 | Down | nucleotide metabolism |
| lmo1835 | | 3213 | 796.5 | 154.1 | -2.329479771 | Down | nucleotide metabolism |
| lmo1836 | | 1092 | 982.41 | 200.145 | -2.266682074 | Down | nucleotide metabolism |
| lmo1837 | | 1281 | 966.885 | 201.965 | -2.22650367 | Down | nucleotide metabolism |
| lmo1838 | | 912 | 594.525 | 129.765 | -2.182567034 | Down | nucleotide metabolism |
| lmo1839 | | 1287 | 1013.45 | 261.57 | -2.028658817 | Down | nucleotide metabolism |
| lmo1840 | | 552 | 497.3 | 217.13 | -1.194918304 | Down | nucleotide metabolism |
| lmo0732 | | 1917 | 17.055 | 37.595 | 1.193536994 | Up | carbohydrate metabolism |
| lmo0733 | | 510 | 97.535 | 422.335 | 2.119163161 | Up | carbohydrate metabolism |
| lmo0734 | | 1005 | 92.805 | 514.69 | 2.392816501 | Up | carbohydrate metabolism |
| lmo0735 | | 672 | 15.22 | 2117.21 | 7.182064004 | Up | carbohydrate metabolism |
| lmo0736 | | 447 | 18.29 | 2406.855 | 7.128821836 | Up | carbohydrate metabolism |
| lmo0737 | | 933 | 22.11 | 2326.49 | 6.766131528 | Up | carbohydrate metabolism |
| lmo0738 | | 1854 | 21.13 | 2706.745 | 6.99395456 | Up | carbohydrate metabolism |
| lmo0739 | | 1374 | 44.785 | 2934.795 | 6.043261389 | Up | carbohydrate metabolism |
| lmo2159 | | 1050 | 27.26 | 58.45 | 1.103049676 | Up | carbohydrate metabolism |
| lmo2160 | | 969 | 23.12 | 52.445 | 1.215227945 | Up | carbohydrate metabolism |
| lmo2761 | | 1455 | 25.765 | 100.215 | 1.904995693 | Up | carbohydrate metabolism |
| lmo2762 | | 303 | 23.005 | 71.48 | 1.801752263 | Up | carbohydrate metabolism |
| lmo2763 | | 1353 | 27.535 | 99.505 | 1.755460775 | Up | carbohydrate metabolism |
| lmo2764 | | 885 | 27.835 | 85.18 | 1.54266118 | Up | carbohydrate metabolism |
| lmo2765 | | 396 | 25.055 | 63.69 | 1.309865484 | Up | carbohydrate metabolism |
