# Supplementary material for: Identification and Characterization of als Genes Involved in D-Allose Metabolism in Lineage II Strain of Listeria monocytogenes
Source: Front Microbiol. 2018 Apr 4;9:621. doi: 10.3389/fmicb.2018.00621 (PMC5893763; doi:10.3389/fmicb.2018.00621)
Supplement: Supplementary file 4 [file Image1.PDF]

A

B

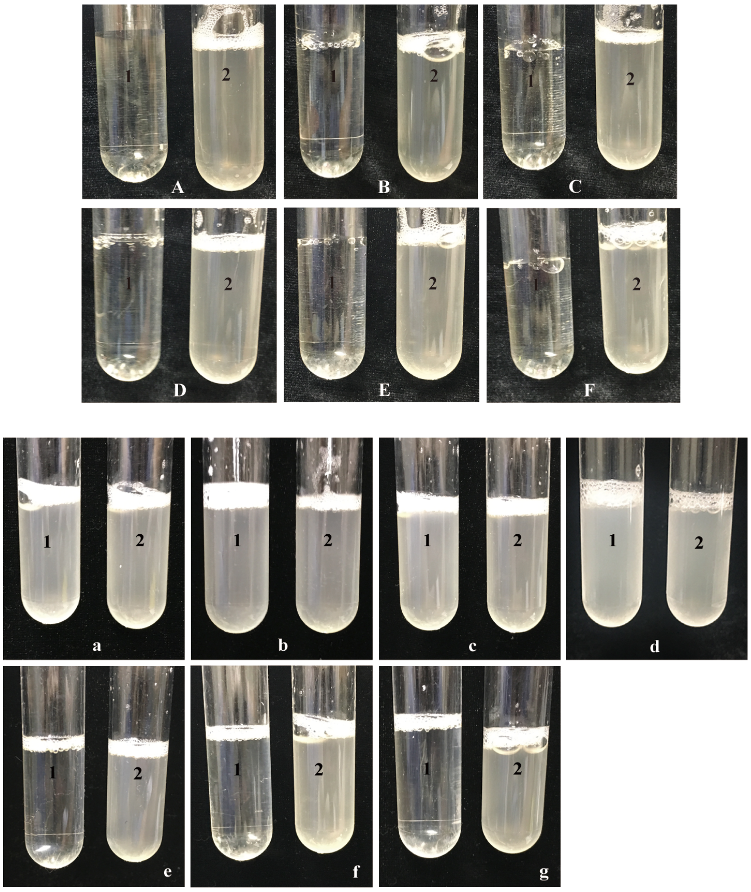

Supplementary Figure 1. The growth of strains and recombinant strains in MWB medium with 0.2% D-allose or D-glucose. (A) Strains of lineage I and III were incubated at 37℃ for 24h, tube1 was displayed as before introducing pAL1, tube2 was displayed as after introducing pAL1. A-F strains: ICDC-LM188, ATCC 19114, ATCC 19115, ATCC 19116, ATCC 19117, ATCC 19118. (B) a-g: RS801 to RS807 were incubated at 37℃ for 24h. Tube1 was 0.2% D-allose MWB medium, tube2 was 0.2% D-glucose MWB medium.
